# The Fungal Exopolysaccharide Galactosaminogalactan Mediates Virulence by Enhancing Resistance to Neutrophil Extracellular Traps

**DOI:** 10.1371/journal.ppat.1005187

**Published:** 2015-10-22

**Authors:** Mark J. Lee, Hong Liu, Bridget M. Barker, Brendan D. Snarr, Fabrice N. Gravelat, Qusai Al Abdallah, Christina Gavino, Shane R. Baistrocchi, Hanna Ostapska, Tianli Xiao, Benjamin Ralph, Norma V. Solis, Mélanie Lehoux, Stefanie D. Baptista, Arsa Thammahong, Robert P. Cerone, Susan G. W. Kaminskyj, Marie-Christine Guiot, Jean-Paul Latgé, Thierry Fontaine, Donald C. Vinh, Scott G. Filler, Donald C. Sheppard

**Affiliations:** 1 Department of Microbiology & Immunology, McGill University, Montreal, Quebec, Canada; 2 Division of Infectious Diseases, LA Biomedical Research Institute at Harbor—UCLA, Torrance, California, United States of America; 3 Department of Immunology and Infectious Diseases, Montana State University, Bozeman, Montana, United States of America; 4 Infectious Disease Susceptibility Program, McGill University Health Centre, Montreal, Quebec, Canada; 5 Department of Microbiology & Immunology, Geisel School of Medicine at Dartmouth, Hanover; 6 Department of Biology University of Saskatchewan, Saskatoon, Saskatchewan, Canada; 7 Department of Pathology Montreal Neurological Hospital Montreal, Quebec, Canada; 8 Aspergillus Unit, Institut Pasteur, Paris, France; 9 David Geffen School of Medicine at University of California, Los Angeles, Los Angeles, California, United States of America; 10 Department of Medicine, McGill University, Montreal, Quebec, Canada; University of Birmingham, UNITED KINGDOM

## Abstract

Of the over 250 *Aspergillus* species, *Aspergillus fumigatus* accounts for up to 80% of invasive human infections. *A*. *fumigatus* produces galactosaminogalactan (GAG), an exopolysaccharide composed of galactose and N-acetyl-galactosamine (GalNAc) that mediates adherence and is required for full virulence. Less pathogenic *Aspergillus* species were found to produce GAG with a lower GalNAc content than *A*. *fumigatus* and expressed minimal amounts of cell wall-bound GAG. Increasing the GalNAc content of GAG of the minimally pathogenic *A*. *nidulans*, either through overexpression of the *A*. *nidulans* epimerase UgeB or by heterologous expression of the *A*. *fumigatus* epimerase Uge3 increased the amount of cell wall bound GAG, augmented adherence *in vitro* and enhanced virulence in corticosteroid-treated mice to levels similar to *A*. *fumigatus*. The enhanced virulence of the overexpression strain of *A*. *nidulans* was associated with increased resistance to NADPH oxidase-dependent neutrophil extracellular traps (NETs) *in vitro*, and was not observed in neutropenic mice or mice deficient in NADPH-oxidase that are unable to form NETs. Collectively, these data suggest that cell wall-bound GAG enhances virulence through mediating resistance to NETs.

## Author Summary

The ubiquitous mold *A*. *fumigatus* is isolated in over 80% of all patients with invasive aspergillosis (IA). *A*. *nidulans* is a relatively non-pathogenic species that rarely causes IA except in patients with chronic granulomatous disease (CGD), a hereditary disease characterized by impaired neutrophil function due to mutations in the NADPH oxidase complex. Here, we demonstrate that one factor underlying the differences in the intrinsic virulence between *A*. *fumigatus* and *A*. *nidulans* is the amount of the exopolysaccharide galactosaminogalactan that is associated with the cell wall of these species. *A*. *fumigatus* produces higher amounts of cell wall-associated galactosaminogalactan and is more resistant than *A*. *nidulans* to neutrophil killing by NADPH-oxidase dependent extracellular traps (NETs). Increasing cell wall-associated galactosaminogalactan in *A*. *nidulans* enhanced resistance to NETs and increased the virulence of this species to the same level as *A*. *fumigatus* in mice with intact NET formation. Collectively, these data suggest that *A*. *nidulans* is more sensitive than *A*. *fumigatus* to NADPH-oxidase dependent NETosis due to lower levels of cell wall-associated GAG.

## Introduction

Invasive aspergillosis (IA) is the most common invasive mold infection in humans. In immunocompromised patients, the inhalation of airborne spores of *Aspergillus* species leads to a necrotizing fungal pneumonia that can disseminate hematogenously to the brain and other organs [1]. Although the genus *Aspergillus* is comprised of over 250 members, *Aspergillus fumigatus* is responsible for more than 80% of invasive aspergillosis cases [2]. On the other hand, *Aspergillus nidulans* is a model, non-pathogenic organism used extensively for the study of eukaryotic cell biology. Interestingly, *A*. *nidulans* is rarely a cause of invasive disease, except in patients with chronic granulomatous disease (CGD), a genetic disorder of the NADPH oxidase system that results in impaired production of reactive oxygen species by phagocytes [3–6]. The predominance of *A*. *fumigatus* as a cause of invasive disease in patients without CGD is not reflected in air or environmental sampling studies in which *A*. *fumigatus* accounts for only a minority of the total *Aspergillus* species recovered [7]. These observations suggest that *A*. *fumigatus* possesses unique virulence traits that enhance its ability to cause human infection. Disruption of a number of putative virulence factors of *A*. *fumigatus* results in attenuated virulence of this species (reviewed in [7,8]). However, none of these factors have been demonstrated to confer increased virulence on less pathogenic *Aspergillus* species such as *A*. *nidulans*. The identification of this type of transferable virulence factor would improve our understanding of the pathogenesis of IA, and could potentially guide the development of novel therapeutic strategies.

Recently, we and others have reported that the secreted and cell wall exopolysaccharide galactosaminogalactan (GAG) is required for normal virulence of *A*. *fumigatus* [9,10]. GAG is an α-1,4-linked linear heteroglycan composed of a variable combination of galactose and N-acetyl-galactosamine (GalNAc). Although the pathways governing GAG synthesis are not fully understood, two UDP-glucose 4-epimerases, Uge5 and Uge3, are required for GAG production. Uge5 mediates the conversion of UDP-glucose to UDP-galactose, while Uge3 is a bifunctional epimerase that can mediate both the interconversion of UDP-glucose to UDP-galactose and of UDP-N-acetylglucosamine to UDP-GalNAc [11]. Deletion of *uge5* results in the production of GAG with a lower galactose content, while deletion of *uge3* completely abrogates GAG synthesis [10,11]. GAG plays a number of roles in host-pathogen interactions [9,10]. This glycan mediates adherence to a variety of substrates, including host cells, and is required for normal biofilm formation [12]. Also, GAG covers the surface of hyphae to conceal β-1,3-glucans from recognition by the pattern recognition receptor dectin-1 leading to decreased pulmonary inflammation [10]. Purified, soluble GAG also induces natural killer (NK) cell-mediated apoptosis of neutrophils *in vitro* [13], and administration of soluble GAG is immunosuppressive through the induction of IL-1RA production [14]. Consistent with the pathogenic function of GAG, a GAG-deficient *A*. *fumigatus* mutant was found to have attenuated virulence in a mouse model of invasive aspergillosis [10]. Synthesis of linear α-1,4-linked GAG has been reported in other *Aspergillus* species, including *A*. *parasiticus* [15], *A*. *niger* [16], and *A*. *nidulans* [17,18], although the quantity of GAG from these species has not been compared. In light of the important role that GAG plays in the virulence of *A*. *fumigatus*, we investigated whether differences in GAG production or composition might contribute to the spectrum of virulence observed among *Aspergillus* species.

## Results

### *A*. *fumigatus* GAG contains higher levels of GalNAc than GAG produced by other *Aspergillus* species

**Fig 1:**
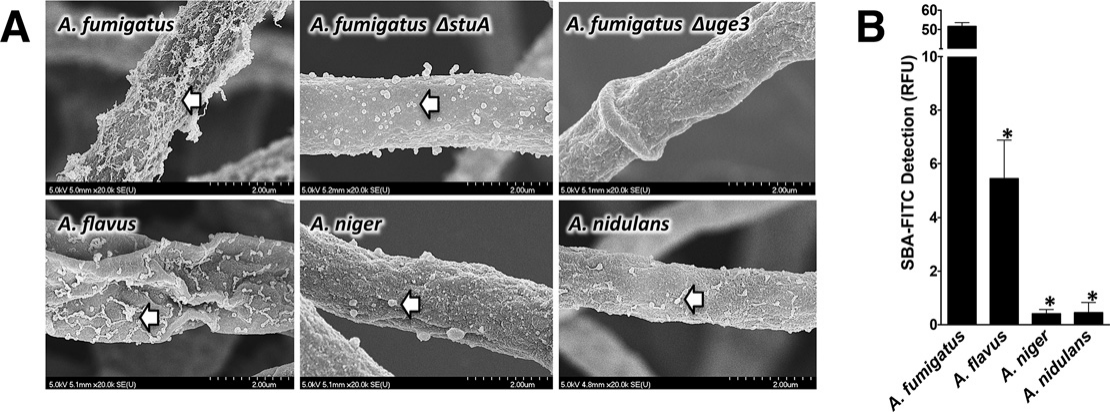
Production of GalNAc-rich GAG correlates with reported virulence of *Aspergillus spp*. (A) Scanning electron micrograph of hyphae of indicated species at 20,000X magnification. Arrows indicate surface decorations associated with cell wall-bound GAG. The GAG deficient *A*. *fumigatus* Δ*uge3* mutant [10] and the *A*. *fumigatus* Δ*stuA* mutant [49] which produces only minimal amounts of GAG are included for comparison purposes. (B) Cell wall GalNAc staining with FITC-conjugated soybean agglutinin (SBA). SBA binding to mature hyphal mats of the indicated species was quantified by fluorometry. Data are represented as mean +/- SEM. * indicates a significant difference between *A*. *fumigatus* and other species, p<0.05 by ANOVA and pairwise comparison.

To determine if different species of *Aspergillus* produce different levels of secreted GAG, we investigated two medically relevant species, *A*. *fumigatus* as well as *A*. *flavus*, which is the second most common *Aspergillus* isolate in IA patients [19]. We also studied the less pathogenic *A*. *niger* and *A*. *nidulans*. When these organisms were grown under GAG-inducing conditions, all species secreted similar amounts of GAG into the medium, as recovered by ethanol precipitation (S1A Fig). However, scanning electron microscopy (SEM) of hyphae from each of these *Aspergillus* species demonstrated dramatic differences in the amount of GAG-associated decorations on the cell wall of hyphae (Fig 1A) [10]. *A*. *fumigatus* displayed abundant cell wall-bound decorations, while the other species more closely resembled the previously described *A*. *fumigatus* Δ*stuA* mutant, which produces very low levels of GAG (Fig 1A) [10]. To confirm that the alterations in cell wall morphology reflected changes in the amount of cell wall-associated GAG, the amount of cell wall-bound GAG was examined by staining hyphae of each of the *Aspergillus* species with the GalNAc specific lectin, soybean agglutinin (SBA) [10,11,20,21]. Staining of cell wall-bound GalNAc by SBA was strongest with *A*. *fumigatus*, followed by *A*. *flavus*, while *A*. *niger* and *A*. *nidulans* exhibited minimal SBA binding (Fig 1B). Importantly, similar levels of hyphal cell wall SBA staining were observed with the commonly used *A*. *nidulans* laboratory strain A26 and three other clinical isolates of this species (S1B and S1C Fig),. Interestingly, the amount of GalNAc-rich GAG and cell wall decorations produced by these species paralleled their frequency of recovery from patients with invasive aspergillosis. Collectively, these results suggest that *Aspergillus* species exhibit significant differences in the amount of cell wall-associated GAG. Moreover, these differences correlate with the reported intrinsic virulence of these species. In light of these findings, *A*. *fumigatus* and *A*. *nidulans* A26 were selected for further study as representatives of highly pathogenic and minimally pathogenic species of *Aspergillus* species that display significant differences in cell wall-bound GAG.

### *A*. *nidulans* produces GalNAc-poor GAG and has impaired biofilm formation as compared with *A*. *fumigatus*

**Fig 2:**
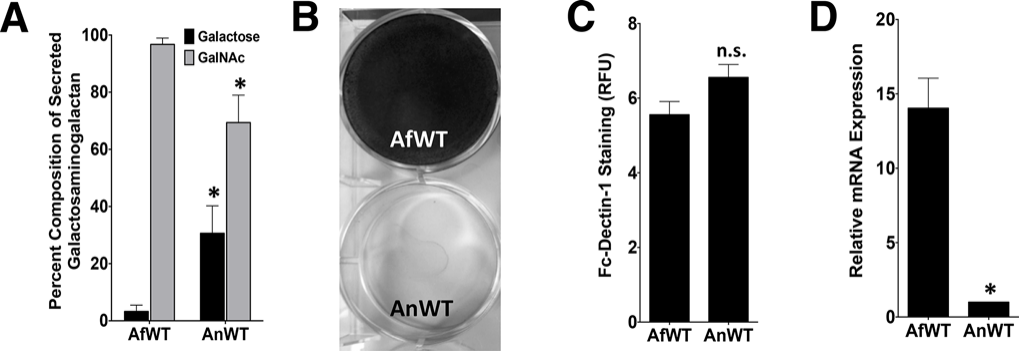
*nidulans* produces GalNAc-poor GAG which is associated with non-adherence. ***A*.** (A) Galactose and GalNAc content of secreted GAG from either *A*. *fumigatus* or *A*. *nidulans* as identified by gas chromatography and quantified by hexose or hexosamine assays. (B) Formation of adherent biofilms on tissue culture-treated polystyrene plates by *A*. *fumigatus* and *A*. *nidulans*. After 24 hours growth, biofilms were washed and visualized by staining with 0.1% crystal violet. (C) Detection of β-1,3-glucan exposure on the surface of hyphae by immunostaining with Fc-dectin-1 antibody by fluorometry. (D) Relative expression of *ugeB* in *A*. *nidulans* and *uge3* in *A*. *fumigatus*, during growth in Brian medium as measured by real-time RT-PCR. Expression of *tef1* from each respective species was used as an internal reference gene. Primer efficiency was verified, and was not different between species (S2E Fig). For all panels: data are represented as mean +/- SEM. AfWT indicates *A*. *fumigatus*, and AnWT indicates *A*. *nidulans*. * indicates a significant difference between *A*. *fumigatus* and *A*. *nidulans*, p<0.05 by Student *t* test or ANOVA with Tukey’s test for pairwise comparison, where applicable.

To test if the degree of cell wall-bound GAG could reflect differences in the composition of GAG produced by *A*. *fumigatus* and *A*. *nidulans*, monosaccharide analysis of secreted GAG from both species was performed by gas chromatography. Secreted GAG produced by *A*. *nidulans* contained significantly less GalNAc and more galactose as compared with *A*. *fumigatus* GAG (Fig 2A).

To test if the production of lower GalNAc-containing GAG by *A*. *nidulans* could affect the known virulence-associated properties of GAG, *A*. *nidulans* was compared with *A*. *fumigatus* with respect to its ability to form adherent biofilm and to mask hyphal β-glucan exposure. *A*. *nidulans* A26 and three clinical isolates of *A*. *nidulans* formed less adherent biofilms (Figs 2B and S1) as compared with *A*. *fumigatus*. In contrast, β-glucan binding by recombinant Fc-dectin-1 was not different between these two species (Fig 2C). These observations suggest that the quantity of cell wall bound GAG as well as the ability of GAG to mediate biofilm formation are dependent on the GalNAc content of GAG. In contrast, β-glucan masking appears to be GalNAc-independent, or at least require a lower amount of this hexosamine.

### *A*. *nidulans* produces GAG with a lower GalNAc content due to low levels of expression of the glucose epimerase UgeB

The synthesis of the GalNAc component of GAG in *A*. *fumigatus* results from the activity of the UDP-glucose 4-epimerase, Uge3 [10,11]. A search of the *A*. *nidulans* genome identified *ugeB* as the gene whose product has the closest homology to *A*. *fumigatus* Uge3 (85% amino acid identity). As with the *A*. *fumigatus uge3* gene [10], deletion of *ugeB* in *A*. *nidulans* resulted in a strain whose hyphae lacked detectable GalNAc by SBA staining (S2A Fig), confirming that Uge3 and UgeB both mediate GalNAc synthesis. Consistent with a previous report that *ugeB* expression is extremely low in *A*. *nidulans* [22], real-time RT-PCR demonstrated that the expression of *ugeB* in *A*. *nidulans* was significantly lower than the expression of *uge3* in *A*. *fumigatus* (Fig 2D), suggesting that the lower GalNAc content of GAG produced by *A*. *nidulans* may be due to the lower expression levels of *ugeB*. As expected, expression of *uge3* was not detected in *A*. *nidulans* (S2B Fig).

### Overexpression of *uge3* or *ugeB* in *A*. *nidulans* increases the GalNAc content and cell wall binding of GAG, as well as enhances biofilm formation

**Fig 3:**
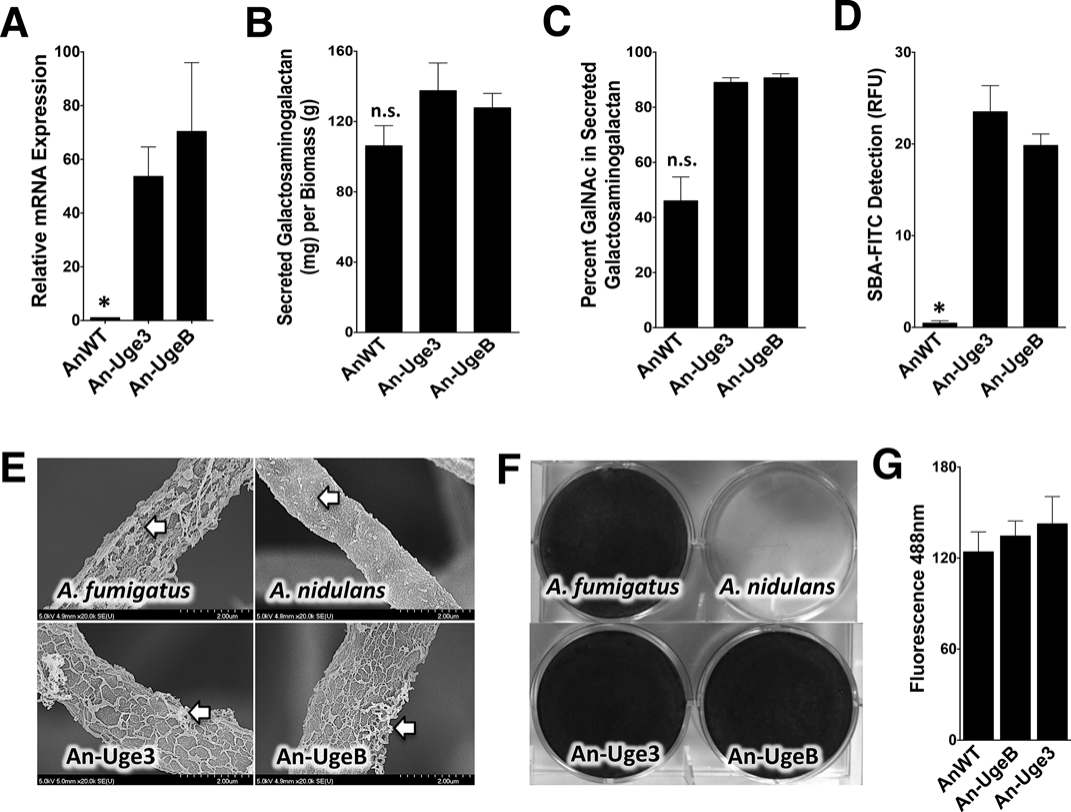
Overexpression of *uge3* or *ugeB* in *A*. *nidulans* increases the GalNAc content of GAG and enhances the formation of adherent biofilms. (A) Relative expression of *uge3* in the An-Uge3 strain and *ugeB* in the An-UgeB strain compared to the expression level of *ugeB* in wild-type *A*. *nidulans* grown in Brian medium and as measured by real-time RT-PCR. (B) Total amount of secreted GAG from the indicated strains. (C) GalNAc content of secreted GAG from the indicated strains as determined by gas chromatography and quantified by hexose or hexosamine assays. (D) Cell wall GalNAc staining with FITC-conjugated soybean agglutinin (SBA). SBA binding to mature hyphal mats of the indicated strains was quantified by fluorometry. (E) Scanning electron micrograph of hyphae of indicated species at 20,000X magnification. Arrows indicate surface decorations associated with cell wall-bound GAG. (F) Formation of adherent biofilms on tissue culture treated polystyrene plates by the indicated strains. After 24 hours growth, biofilms were washed and visualized by staining with 0.1% crystal violet. (G) Detection of β-1,3-glucan exposure on the surface of hyphae by immunostaining with Fc-dectin-1 antibody labeled with FITC secondary antibody and quantified by fluorometry at 495 nm. For all panels: An-Uge3 indicates the *A*. *nidulans* overexpressing *uge3* strain; An-UgeB indicates the *A*. *nidulans* overexpressing *ugeB* strain; and AnWT indicates wild type *A*. *nidulans*. Data are represented as mean +/- SEM and * indicates a significant difference between *A*. *nidulans*, and both overexpression strains, p<0.05 by ANOVA with Tukey’s test for pairwise comparison.

To test the hypothesis that the low GalNAc content of *A*. *nidulans* GAG results from low expression of *ugeB*, strains of *A*. *nidulans* were constructed in which the *A*. *fumigatus uge3* or *A*. *nidulans ugeB* genes were expressed under the constitutively active *gpdA* promoter to produce strains An-Uge3 and An-UgeB, respectively (Fig 3A). Overexpression of either *uge3* or *ugeB* had no significant effect on total secreted GAG production (Fig 3B). However, increased expression of either gene resulted in an increase in the GalNAc content of secreted GAG (Fig 3C) to levels similar to that found in *A*. *fumigatus*. Even more dramatically, overexpression of either gene markedly increased the amount of cell wall-bound GAG as detected by SBA lectin binding (Fig 3D) and SEM to levels indistinguishable from *A*. *fumigatus* (Fig 3E). These data suggest that the GalNAc content of GAG is important in determining the amount of GAG that binds to the hyphal cell wall.

Augmenting the GalNAc content of GAG in *A*. *nidulans* resulted in a marked increase in biofilm formation (Fig 3F), but had no effect on the size or germination of the conidia (S2C and S2D Fig), β-1,3-glucan masking (Figs 3G and S2F), or hyphal growth rate (S2G Fig). Collectively, these data suggest that increasing GalNAc content of *A*. *nidulans* GAG by overexpressing either the native *A*. *nidulans ugeB* or *A*. *fumigatus uge3* gene resulted in a strain of *A*. *nidulans* that resembled *A*. *fumigatus*, *in vitro*.

### Increasing the GalNAc content of *A*. *nidulans* GAG increases the virulence of *A*. *nidulans*

**Fig 4:**
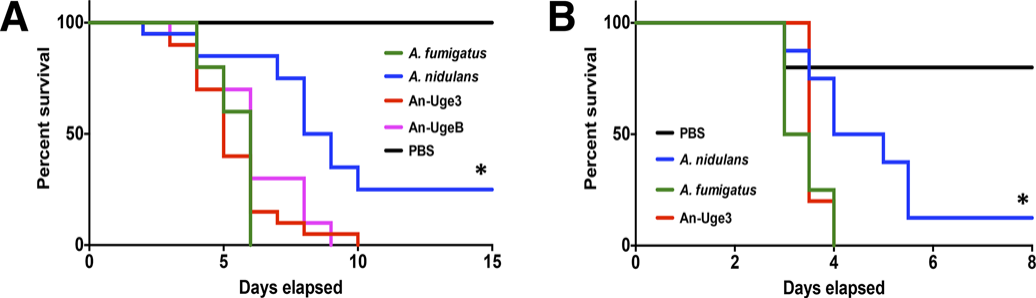
Overexpression of *uge3* in *A*. *nidulans* increases virulence in mice. (A) Survival of corticosteroid treated BALB/c mice infected with the indicated conidial species and strains. Data represent the results from two independent experiments for total N = 20 for *A*. *nidulans* or An-Uge3; N = 10 for *A*. *fumigatus* or An-UgeB; N = 8 for PBS control. (B) Survival of corticosteroid treated C57BL/6 mice infected with the indicated conidial species and strains. Five or more mice were infected for each group. * indicates a significant difference in survival of *A*. *nidulans* compared with *A*. *fumigatus*, An-Uge3, and An-UgeB overexpression strains as determined by the Mantel-Cox log-rank test with pairwise comparison applying Bonferroni correction.

To determine if the increase in cell wall-bound GalNAc-rich GAG resulting from overexpression of either *uge3* or *ugeB* could result in increased virulence, corticosteroid-treated Balb/c and C57BL/6 mice were infected intranasally with wild-type *A*. *fumigatus*, wild-type *A*. *nidulans*, An-Uge3, or An-UgeB. As expected, mice infected with wild-type *A*. *nidulans* had a longer median survival than those infected with *A*. *fumigatus* (Fig 4A and 4B). Also, 10–25% of mice infected with wild-type *A*. *nidulans* survived to the end of the experiment, whereas none of the animals infected with *A*. *fumigatus* survived. In contrast, the onset of death, median survival and overall mortality of mice infected with An-Uge3 or An-UgeB were similar to mice infected with *A*. *fumigatus*. Similar findings were observed in Balb/c (Fig 4A) and C57BL/6 mice (Fig 4B), suggesting these differences in virulence were not mouse strain specific. Thus, increasing the GalNAc content of GAG in *A*. *nidulans* by increasing expression of a heterologous or endogenous GalNAc epimerase significantly enhanced the virulence of this minimally pathogenic *Aspergillus* species.

### Increasing the GalNAc content of *A*. *nidulans* GAG resulted in increased tissue invasion

**Fig 5:**
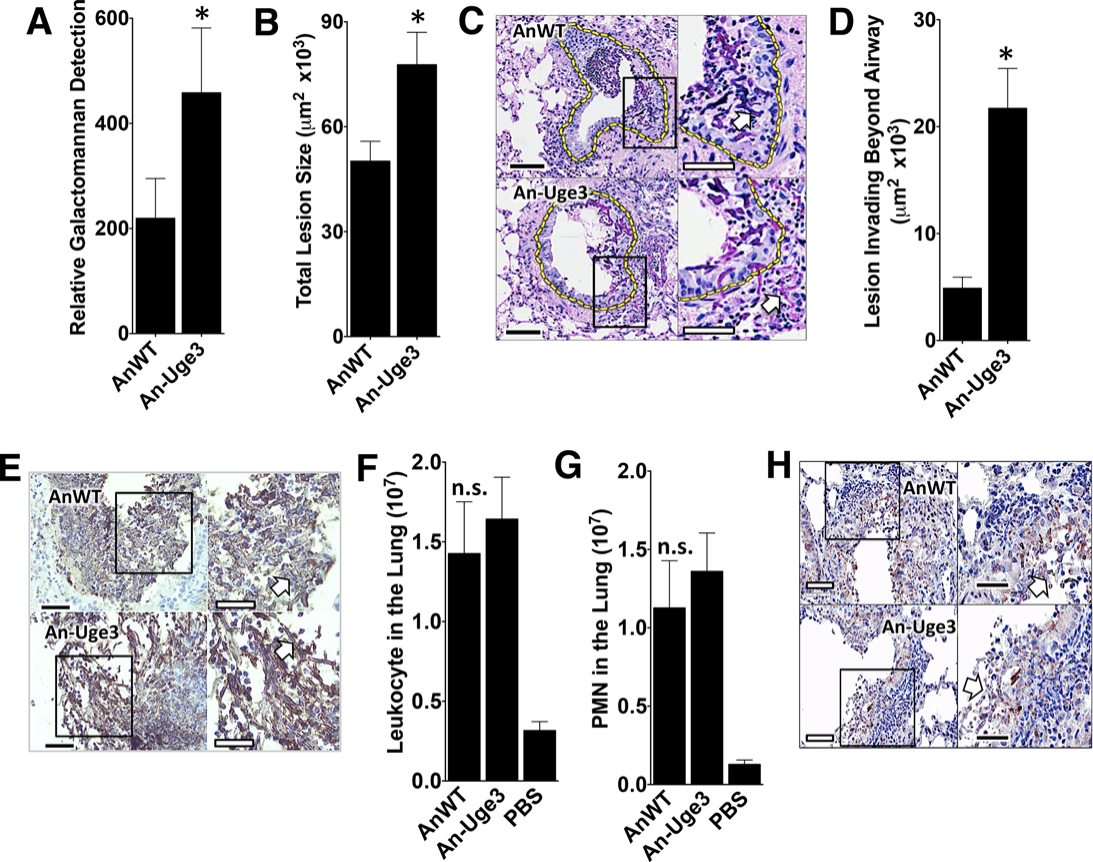
Increase in virulence of the *A*. *nidulans* strain overexpressing *uge3* is associated with increase in fungal burden and pulmonary tissue invasion. (A) Pulmonary fungal burden measured by relative galactomannan content in the lungs of mice infected with the indicated strains, N = 10 Balb/c mice for each fungal strain. (B) Total fungal lesion size as determined by morphometric analysis of lung histopathology for the indicated strains, N = 4 Balb/c mice for each fungal strain. (C) Pulmonary histopathology sections from Balb/c mice infected with indicated strains and stained with PAS for visualization of fungi. The yellow dotted line indicates the limit of the airway used for morphometric analysis. White arrow indicates fungal elements outside the airway and invading into pulmonary tissues. Scale bar represents 100 μm (black) or 50 μm (white). (D) Lesion invasion beyond the airway as determined by morphometric analysis of infected mouse lung histopathology for the indicated strains, N = 4 Balb/c mice for each fungal strain. (E) Pulmonary histopathology sections from Balb/c mice infected for 4 days with the indicated strains and labeled with an anti-GAG antibody to visualize GAG on hyphae. Arrow indicates hyphae. Scale bar represents 80 μm (black) or 30 μm (white). (F) Total pulmonary leukocytes from mice 4 days after infection with the indicated strains as measured by CD45 detection by flow cytometry. N = 10 Balb/c mice for each fungal strain, N = 5 for PBS control. (G) Total pulmonary neutrophils from of mice 4 days after infection with the indicated strains as measured by Ly6G^+^, CD11b^high^, and CD11c^low^ detection using flow cytometry. N = 10 Balb/c mice for each fungal strain, N = 5 Balb/c mice for PBS control. (H) Pulmonary histopathology sections from Balb/c mice 4 days after infection with the indicated strains and labeled with anti-caspase-3 antibody to visualize host cells undergoing apoptosis. Arrow indicates hyphae. Scale bar represents 100 μm (black) or 50 μm (white). For all panels: An-Uge3 indicates the *A*. *nidulans* overexpressing *uge3* strain and AnWT indicates wild type *A*. *nidulans*. For panel A, B, D, F, and G, data are represented as median with interquartile ranges and * indicates a significant difference between *A*. *nidulans* and the An-Uge3 strain, p<0.05 by Kruskal-Wallis test with Dunn’s test for pairwise comparison.

To examine the mechanisms underlying the increased virulence seen with overexpression of *uge3* or *ugeB* in *A*. *nidulans*, corticosteroid-treated mice were infected with wild-type *A*. *nidulans* or An-Uge3 and their lungs examined after 4 days of infection. The pulmonary fungal burden of mice infected with An-Uge3 was significantly higher than in mice infected with wild-type *A*. *nidulans*, as measured by galactomannan content (Fig 5A) and quantitative morphometric analysis of histopathology sections (Fig 5B). Histopathologic examination of lungs from these mice revealed striking differences in the degree of pulmonary invasion between wild-type *A*. *nidulans* (Fig 5C, top) and An-Uge3 (Fig 5C, bottom). Hyphae of wild-type *A*. *nidulans* were largely restricted to the airway lumen with minimal penetration into the pulmonary parenchyma. In contrast, hyphae of the An-Uge3 strain were markedly more invasive and penetrated significantly deeper into pulmonary tissues. Quantitative morphometric analysis of histopathology sections confirmed that hyphae of the An-Uge3 strain invaded much further from the airways than the parental *A*. *nidulans* strain (Fig 5D). Importantly, the magnitude of tissue invasion by the An-Uge3 strain (5-fold (Fig 5D)) was much greater than the increase in total lesion size (1.5-fold (Fig 5B)), suggesting that the increased invasiveness was not simply a reflection of increased lesion size. In fact, in mice infected with the An-Uge3 overexpression strain, only 2 out of 51 lesions remained confined within the airway lumen compared to 18 out of 51 lesions from mice infected with wild-type *A*. *nidulans*. Further histological examination by immunohistochemistry with anti-GAG antibody revealed that the hyphal surface of An-Uge3 hyphae in these lesions was associated with increased cell wall-bound GAG, consistent with *in vitro* observations (Fig 5E). These results suggest that increasing the GalNAc content of GAG in *A*. *nidulans* increases cell wall-associated GAG, and enhances the growth of hyphae within pulmonary tissues.

Cell wall-associated GAG has been reported to modulate immune responses through the masking of hyphal PAMPs [10]. Additionally, soluble GAG has been found to directly induce neutrophil apoptosis and the induction of IL-1RA production by macrophages [9,14]. Multiple parameters of the host inflammatory response were therefore analyzed in lungs of infected mice to determine if An-Uge3 induced a different response than wild-type *A*. *nidulans*. No significant differences were observed between mice infected with *A*. *nidulans* or the An-Uge3 strain with respect to total leukocyte (Fig 5F) or neutrophils (Fig 5G) in the lungs of infected mice. Analysis of the bronchoalveolar lavage fluid also revealed no significant differences between mice infected with *A*. *nidulans* or the An-Uge3 strain with respect to total leukocytes or neutrophils, (S3A and S3B Fig); or total lung myeloperoxidase, TNF-α, IL-1β, IL-17 and IL-1RA levels (S3C–S3G Fig). In addition, no differences in the number of live or dead leukocytes or neutrophils were found between mice infected with wild-type *A*. *nidulans* or the An-Uge3 strain (S3H and S3I Fig). Further, histopathological examination of fungal lesions did not identify any differences in leukocyte nuclear fragmentation surrounding fungal lesions suggestive of differences in apoptosis (S3J Fig), and immunohistochemical staining for caspase-3 did not demonstrate differences between lesions resulting from these two strains (Fig 5H). Taken together, these results suggest that altering the amount of cell wall-bound GAG in *A*. *nidulans* does not alter virulence by altering the host inflammatory response.

### Increasing cell wall-associated GAG enhances resistance to neutrophil killing *in vitro*

**Fig 6:**
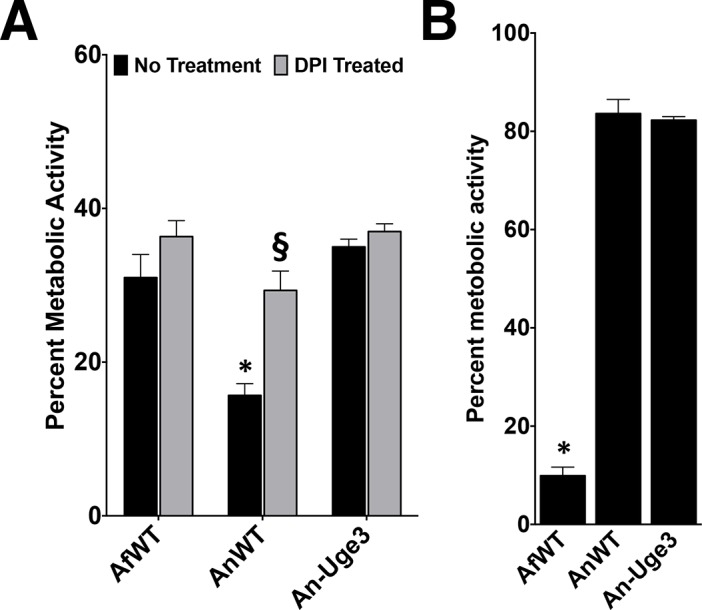
Increasing cell wall-associated GAG enhances the resistance of *A*. *nidulans* to NADPH oxidase-dependent neutrophil extracellular traps (NETs) but not reactive oxygen species. (A) Fungal injury by primary human neutrophils (PMN) either untreated (black bars) or treated with the NADPH oxidase inhibitor diphenyleneiodonium (DPI) (gray bars). (B) Susceptibility of the indicated fungal strains to injury after treatment with 3.2 mM hydrogen peroxide. * indicates a significant difference between *A*. *nidulans* and *A*. *fumigatus* or An-Uge3 strains, p<0.05 by ANOVA with Tukey’s test for pairwise comparison. § indicates a significant difference in killing of *A*. *nidulans* between DPI-treated and untreated neutrophils, p<0.05 by ANOVA with Tukey’s test for pairwise comparison.

Increasing the GalNAc content of GAG in *A*. *nidulans* markedly increased the amount of polysaccharide bound to the hyphal surface and was associated with increased fungal growth and invasion *in vivo*. In light of these findings, we hypothesized that cell wall-associated GAG might function like a capsule and mediate resistance to neutrophil damage during infection. We therefore examined the ability of primary human neutrophils to damage hyphae of *A*. *fumigatus*, *A*. *nidulans* and the An-Uge3 strain. Consistent with our observations *in vivo*, *A*. *nidulans* was more susceptible to damage by neutrophils than *A*. *fumigatus* or the An-Uge3 strain (Fig 6A), suggesting that cell wall-associated GAG enhances resistance to neutrophil killing.

### Increasing cell wall-associated GAG enhances resistance to ROS-independent, NADPH oxidase-dependent neutrophil damage

To probe the mechanism underlying the enhanced resistance of GAG-producing *Aspergillus* to neutrophil-mediated damage, primary human neutrophils were treated with the NADPH oxidase inhibitor, diphenylene iodonium (DPI), and incubated with the fungal strains. Differences in fungal strain susceptibility to neutrophil mediated damage were lost when neutrophils were treated with DPI (Fig 6A). This observation suggests that the cell wall-associated GAG mediates resistance to NADPH oxidase-dependent neutrophil killing. Since NADPH oxidase mediates production of reactive oxygen species, hyphal susceptibility to hydrogen peroxide was examined. Surprisingly. *A*. *nidulans* and the An-Uge3 strain were equally resistant to oxidative stress and significantly more resistant than *A*. *fumigatus* (Fig 6B). These findings suggest that cell wall-associated GAG mediates resistance to NADPH oxidase-dependent neutrophil killing, but not by directly enhancing resistance to reactive oxygen species.

### Increasing cell wall-associated GAG enhances resistance to neutrophil extracellular traps (NETs)

**Fig 7:**
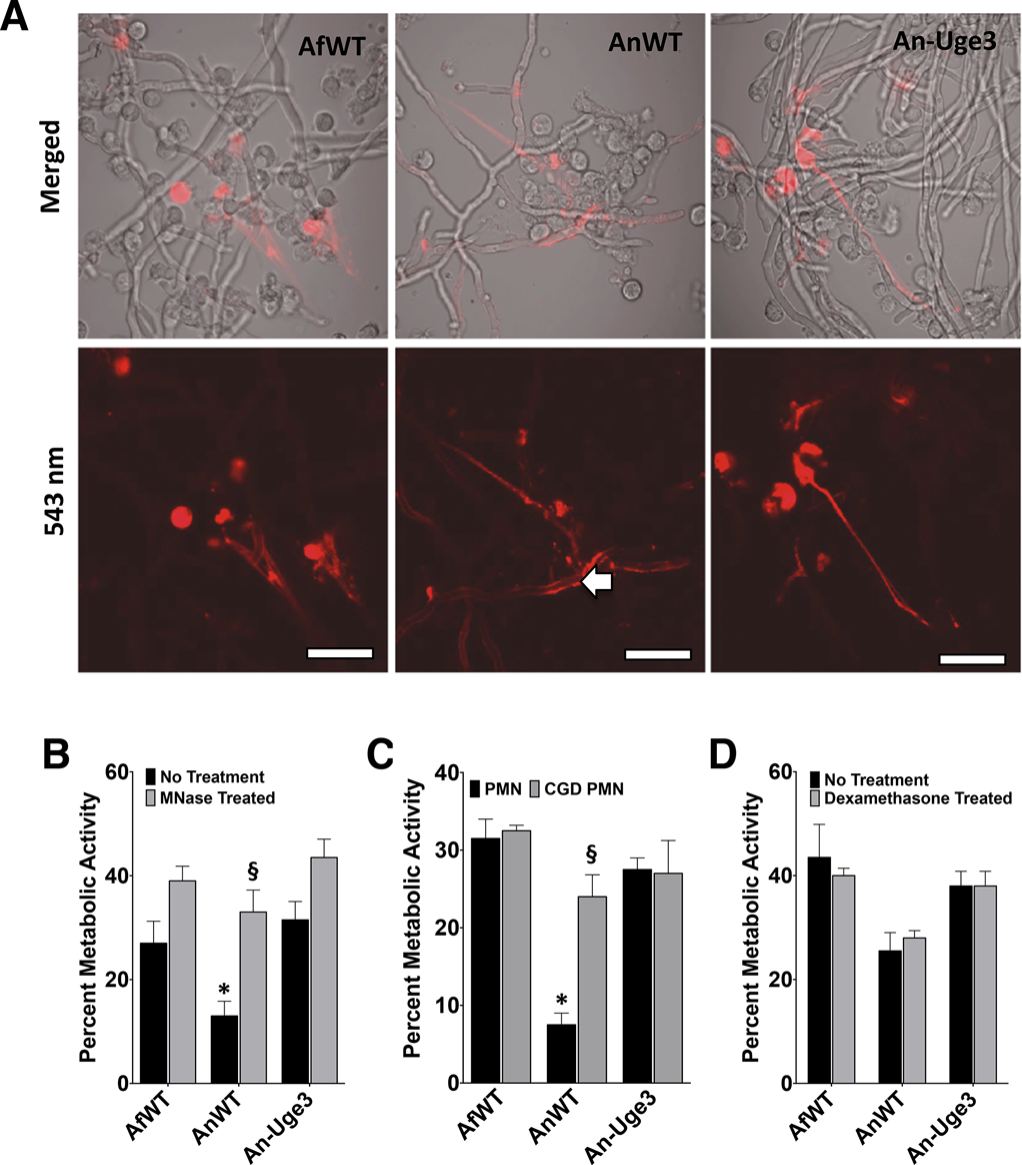
Inhibition or disruption of NETs attenuates the susceptibility of *A*. *nidulans* to killing by human neutrophil-mediated killing. (A) Neutrophil extracellular traps formation by primary human PMN as visualized by the DNA intercalating agent propidium iodide. Arrows indicate the increased binding of propidium iodide stained NETs on the surface of wild-type *A*. *nidulans* hyphae. Images were acquired using a 543 nm laser and detected by confocal microscopy at 600X magnification with 4X digital zoom. Scale bar represents 10 μm. (B) Susceptibility of fungal strains to injury by PMNs in the presence (gray bars) or absence (black bars) of micrococcal nuclease (MNase). (C) Susceptibility of fungal strains to injury by PMNs from healthy donor (black bars) or from a CGD patient (grey bars). (D) Susceptibility of fungal strains to injury by PMNs pre-treated with 10 μM dexamethasone (grey bars) or untreated (black bars). * indicates a significant difference between *A*. *nidulans* and *A*. *fumigatus* or An-Uge3 strains, p<0.05 by ANOVA with Tukey’s test for pairwise comparison. § indicates significant difference treatment groups of PMNs co-incubated with *A*. *nidulans*, p<0.05 by ANOVA with Tukey’s test for pairwise comparison. All data are represented as mean +/- SEM.

**Fig 8:**
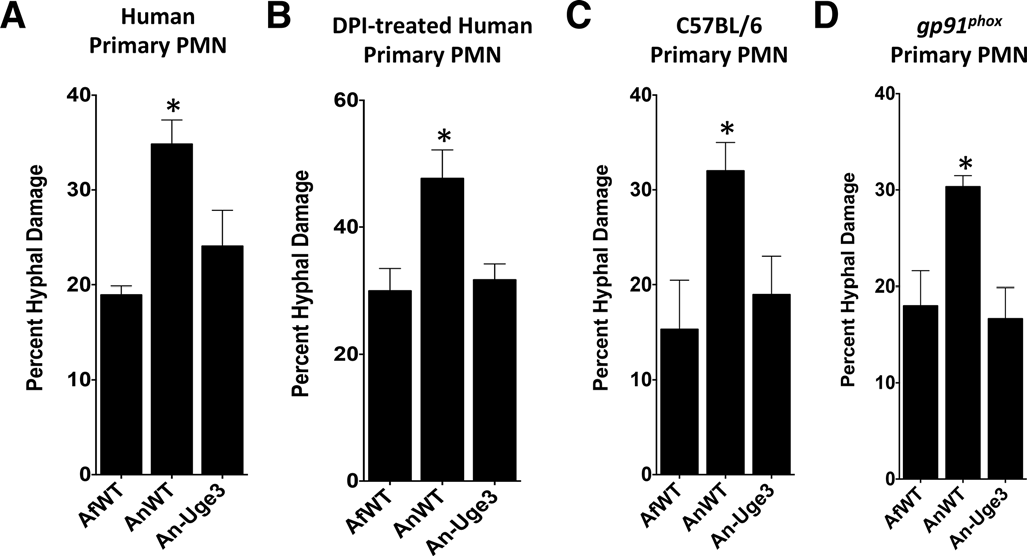
GAG-mediated resistance to neutrophil killing is dependent on neutrophil lysate content. Susceptibility of fungal strains to injury by lysates derived from, (A) primary human neutrophils, (B) primary human neutrophils treated with DPI., (C) primary C57BL/6 mouse neutrophils or *gp91* ^*phox*^ deficient (CGD) mouse neutrophils. * indicates a significant difference between *A*. *nidulans* and *A*. *fumigatus* or An-Uge3 strains, p<0.05 by ANOVA with Tukey’s test for pairwise comparison.

In addition to mediating the production of reactive oxygen species that directly injure fungi, phagocyte NADPH oxidase also mediates antifungal host defense by inducing the coordinated release of DNA and antimicrobial peptides from neutrophil granules to form neutrophil extracellular traps (NETs) [23,24]. NADPH-oxidase dependent NET formation has been reported in response to both *A*. *fumigatus* and *A*. *nidulans*, *in vitro* and *in vivo* [25–28]. To determine if NET formation correlated with the differences in GAG-dependent susceptibility of strains to neutrophil killing, primary neutrophils were co-cultured with *A*. *fumigatus*, *A*. *nidulans* and An-Uge3 and examined for NET formation (Fig 7A). Nuclear de-condensation and the formation of propidium iodide staining NETs were observed during co-culture with all three organisms (Fig 7A). Although the number of neutrophils undergoing NETosis was not different in response to the different fungal strains, more propidium iodide positive NETs were bound by hyphae of wild-type *A*. *nidulans* compared with *A*. *fumigatus* or An-Uge3 (Fig 7A). Treatment of primary human neutrophils with micrococcal nuclease (MNase), which degrades DNA, prevented the formation of NETs as previously reported [29], and reduced the differences in susceptibility to neutrophil-mediated damage among the three *Aspergillus* strains (Fig 7B), mirroring the effects of DPI treatment. Similarly, neutrophils isolated from a patient with CGD failed to form NETs, and did not exhibit enhanced killing of *A*. *nidulans* relative to *A*. *fumigatus* or An-Uge3 (Fig 7C), reproducing the effects of DPI and MNase treatment. Consistent with the differences in strain virulence seen in the corticosteroid-treated mouse model, corticosteroid treated primary neutrophils formed NETs and retained their ability to kill wild-type *A*. *nidulans* to a greater degree than *A*. *fumigatus* or An-Uge3, (Fig 7D). Finally, wild-type *A*. *nidulans* was also found to be more susceptible to primary neutrophil lysates than *A*. *fumigatus* or the An-Uge3 strain (Fig 8A), confirming that cell wall-bound GAG directly mediates resistance to damage by neutrophil contents contained within NETs. Interestingly, similar patterns of differential susceptibility to damage were also seen with lysates collected from DPI-treated human neutrophils, as well as primary neutrophils from C57BL/6 mice and primary neutrophils from NADPH-oxidase deficient mice (Fig 8C and 8D). These observations suggest that, under these conditions, neutrophil NADPH oxidase exerts its antifungal activity largely by orchestrating release of neutrophil intracellular contents, rather than influencing their composition or activity. Collectively these data suggest that cell wall-bound GAG enhances resistance to NADPH oxidase-dependent neutrophil extracellular damage by directly increasing resistance to NETs.

**Fig 9:**
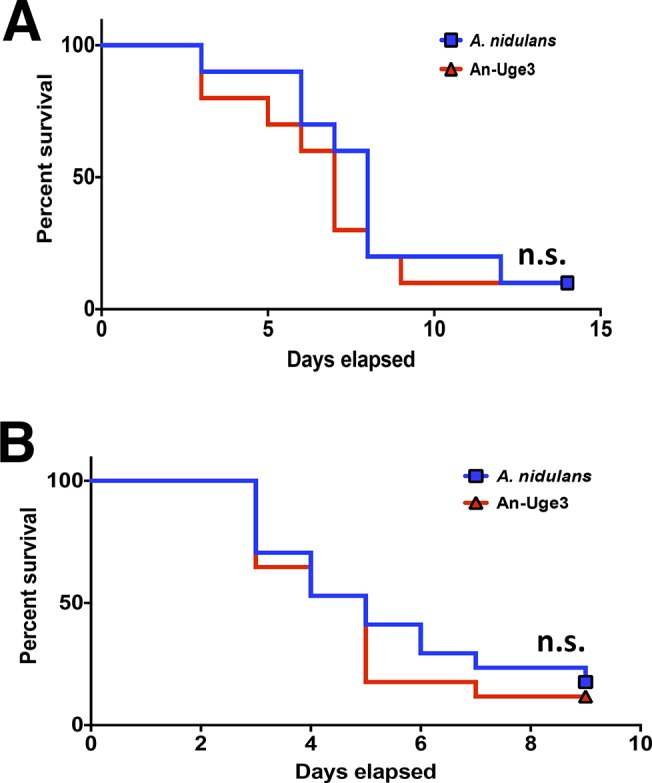
GAG mediated enhancement of *A*. *nidulans* virulence requires functional leukocyte NADPH oxidase. (A) Survival of leukopenic mice infected with either *A*. *nidulans* or An-Uge3 conidia. N = 9 per infection group. (B) Survival of *gp91* ^*phox*^ deficient mice lacking functional NADPH oxidase infected with either *A*. *nidulans* or An-Uge3 conidia. N = 17 per infection group. For all panels: An-Uge3 indicates the *A*. *nidulans* overexpressing *uge3* strain; AnWT indicates wild type *A*. *nidulans*; and AfWT indicates wild type *A*. *fumigatus*. n.s. indicates no significant difference in survival of *A*. *nidulans* compared with the An-Uge3, strain as determined by the Mantel-Cox log-rank test with pairwise comparison applying Bonferroni correction.

To test if GAG-mediated resistance to NETs underlies the increased virulence of the An-Uge3 strain (Fig 4), we compared the virulence of wild-type *A*. *nidulans* and the An-Uge3 strain in two groups of mice that cannot make NETs in response to *Aspergillus* infection: neutropenic mice and *gp91* ^*phox*^ deficient mice, that lack functional NADPH oxidase [26]. Consistent with our in vitro findings, increasing cell wall-associated GAG expression in the An-Uge3 strain did not increase *A*. *nidulans* virulence in either of these NET deficient mice (Fig 9A and 9B). These data strongly suggest that the expression of cell wall-bound GAG by *Aspergillus* mediates virulence through enhancing resistance of hyphae to NADPH oxidase-dependent NET formation.

## Discussion

This study establishes a role for cell wall-associated GAG in mediating resistance to killing of *Aspergillus* by NETs and provides the first example of a virulence factor of *A*. *fumigatus* that is able to mediate enhanced virulence when expressed in a less pathogenic *Aspergillus* species. GAG has been reported to mediate a number of functions *in vitro* that could influence virulence, including host cell adherence, masking of β-glucan, the modulation of host immune responses and the induction of neutrophil apoptosis [10,13,14,34]. While determining the contribution of each of these mechanisms to virulence is challenging, the present study provides some insights into this question. First, β-glucan masking was not different among *A*. *fumigatus A*. *nidulans* and the An-Uge3 strains in our study, suggesting that differences in β-glucan masking and dectin-1 activation do not contribute to the difference in virulence between these strains. Second, the similar levels of virulence exhibited by wild-type *A*. *nidulans* and the An-Uge3 strain in neutropenic and NADPH-oxidase deficient mice suggests that increased biofilm formation by the overexpression strain does not play a significant role in virulence. Finally, increasing the amount of cell wall-bound GAG enhanced *Aspergillus* virulence in the absence of any detectable difference in inflammation or immune response, including pulmonary IL-1RA levels, neutrophil recruitment to site of infection, and the induction of neutrophil apoptosis, which have been observed during treatment with soluble GAG [13,14]. The failure to observe changes in these responses in the present study likely reflects the fact that these effects of GAG were observed in response to treatment with soluble GAG. In the experiments reported here, the amount of shed, soluble GAG was not different between the three strains of *Aspergillus* and only the quantity of cell wall-bound GAG differed among these strains.

Multiple lines of evidence suggest that increased cell wall-associated GAG production augments virulence in non-neutropenic mice through enhancing resistance to neutrophil contents released as extracellular traps. The higher levels of cell wall-bound GAG in *A*. *fumigatus* and An-Uge3 increased resistance to damage by neutrophils and by neutrophil lysates, but not by toxic reactive oxygen species. Moreover, differences in susceptibility to neutrophil mediated damage among *A*. *fumigatus*, wild-type *A*. *nidulans*, and the An-Uge3 strain were not seen with neutrophils deficient in NETosis, isolated either from a patient with CGD, or healthy human neutrophils treated with two inhibitors of NETosis (DPI and MNase). Finally, the increased virulence of the An-Uge3 strain was lost in mice with defective NADPH-oxidase. These data are in agreement with multiple prior studies examining the interactions of *A*. *fumigatus* and *A*. *nidulans* with neutrophils. A previous report suggested that killing of *A*. *nidulans* by human neutrophils occurs predominately via a non-oxidative mechanism [35]. Similarly, while susceptibility of *A*. *nidulans* to killing by NETs has been observed [26], *A*. *fumigatus* growth was found to be only minimally inhibited by NET formation [27]. Our findings suggest that differences in the expression of cell wall-associated GAG between *A*. *nidulans* and *A*. *fumigatus* account for the differential susceptibility to NETs reported in these two studies.

Collectively, the results of these experiments suggest a model in which cell wall-bound GalNAc-rich GAG functions as an extracellular capsule to enhance resistance to NETs, analogous to bacterial capsular exopolysaccharide [11,36–38]. Although the mechanism by which exopolysaccharide mediates resistance to NETs has not yet defined, the increased binding of NETs to *A*. *nidulans* suggests that GAG may directly inhibit NET binding to hyphae. We have previously reported that the GalNAc component of GAG undergoes partial deacetylation, rendering GAG a cationic glycan [39]. It is likely that this positive charge inhibits binding of cationic antimicrobial peptides and histones within NETs resulting in increased resistant to NETosis. A similar mechanism of resistance to cationic antimicrobial peptides by exopolysaccharide-mediated electrostatic repulsion has been described in *Staphylococcus epidermidis* [40]. Further work to clarify this mechanism is clearly required.

Why has *A*. *fumigatus* evolved this change in the composition of an exopolysaccharide and the resulting increase in cell wall-associated GAG? As *A*. *fumigatus* is an environmental organism and only an incidental opportunistic pathogen of immunocompromised hosts, we speculate that selection for GalNAc-rich GAG was mediated through environmental pressures that are unique to *A*. *fumigatus*. One hypothesis is that the production of a capsule-like hyphal sheath could offer protection against competing microorganisms in the complex microbial environment of decomposing organic matter, where *A*. *fumigatus* is commonly found. Studies comparing the production and composition of GAG in strains of different environmental origin may be helpful in shedding light on this question.

Invasive aspergillosis can develop in non-neutropenic patients who are immunocompromised due to corticosteroid or other immunosuppressive therapies, as well as in patients with quantitative or qualitative defects in neutrophil function. The findings of this study are most relevant to invasive aspergillosis in corticosteroid-treated or other non-neutropenic patients in whom NET production is preserved. It is likely that there are other factors that contribute to the spectrum of intrinsic virulence of *Aspergillus* species in different hosts. For example, GAG-mediated resistance to NETs clearly plays no role in the pathogenesis in invasive aspergillosis associated with neutropenia. In CGD patients, who are distinctly more susceptible to *A*. *nidulans*, the inability to form NETs may have a greater effect on increasing susceptibility to infection with *A*. *nidulans* relative to *A*. *fumigatus*. However, further studies comparing the pathogenesis of *A*. *fumigatus* and *A*. *nidulans* infection in CGD mice, or in other models of invasive aspergillosis, are required to understand the role that GAG and other fungal factors play in the pathogenesis of this disease.

Although our data suggests that cell wall-bound GAG may contribute to differences in intrinsic virulence in species other than *A*. *fumigatus* and *A*. *nidulans*, more work is needed to confirm this hypothesis. The biochemical, antibody and lectin studies used to quantify GAG were optimized with *A*. *fumigatus* and it is therefore possible that some differences in GAG composition and structure other than GalNAc content could be missed by these techniques. Also, while we observed similar low levels of cell wall-bound GAG in multiple laboratory and clinical isolates of *A*. *nidulans* strains, a larger screen of clinical and environmental isolates will be required to more firmly establish the association between GalNAc-rich, cell wall-bound GAG and intrinsic virulence. In addition, further mechanistic studies modulating GAG composition in these other species are required to confirm that the observations made in *A*. *nidulans* are applicable to other species that produce GalNAc-poor GAG.

Modulating the composition of a single exopolysaccharide significantly enhanced the virulence of a relatively non-pathogenic *Aspergillus* species in corticosteroid-treated mice through enhancing resistance to NETs. This study highlights the importance of GAG as a key virulence factor of *A*. *fumigatus* in this population and suggests that targeting this exopolysaccharide may be an effective antifungal approach.

## Materials and Methods

### Fungal strains and growth conditions

*A*. *nidulans* strain A26 (Fungal Genetics Stock Center, Kansas City, MO, USA) was used as the parent wild-type strain for all molecular manipulations. Other strains used in this study include *A*. *fumigatus* strain Af293 (a generous gift from P. Magee, University of Minnesota, St. Paul, MN, USA), the *A*. *fumigatus* Δ*uge3* mutant [10], Δ*stuA* mutant [41] and clinical isolates of *A*. *flavus* and *A*. *niger* (obtained from the McGill University Health Center, Montreal, QC, Canada) and *A*. *nidulans* isolates from CGD patients (generous gifts from A. Warris and S. Henriett, Radboud University Medical Center, Nijmegen, The Netherlands, and Adrian Zelazny US National Institute of Health, USA). Unless otherwise noted, *A*. *fumigatus* strains and *A*. *flavus* were maintained on YPD agar (Fisher Scientific), *A*. *niger* on potato-dextrose agar (Fisher Scientific), and *A*. *nidulans* strains on Aspergillus minimum medium agar [10] at 37°C. For growth in liquid medium, Brian medium [9] and phenol red-free RPMI 1640 (Wisent, Inc.) were used as indicated. All growth media for *A*. *nidulans* strains were supplemented with biotin (Fisher Scientific).

### Molecular and genetic manipulations

Heterologous overexpression of *uge3* and endogenous overexpression of *ugeB* in *A*. *nidulans* was performed as previously described [42,43], with minor modifications. The open reading frames of *uge3* or *ugeB* were amplified by PCR from genomic DNA of *A*. *fumigatus* or *A*. *nidulans*, respectively. The resulting PCR products were cloned downstream of the constitutive *gpdA* promoter in plasmid pGFP-Phleo [42] [44] by replacing the GFP coding regions with either *uge3* or *ugeB* to yield plasmids pUge3-OX and pUgeB-OX, respectively (S1 Table). *A*. *nidulans* was then transformed by spheroplasting, as previously described [45]. Gene overexpression was confirmed by real time RT-PCR [11].

### Mutant characterization

Purification and analysis of GAG was performed as previously described [11]. Hyphae were grown for 72h, and GAG was precipitated from culture supernatants with 2.5 volumes of ethanol. The GalNAc-rich insoluble fraction was collected by filtration on Nylon membrane and washed with 60% ethanol (2.5 volume). Other volumes of ethanol precipitation was tested with similar results. Composition of GAG was determined by gas chromatography after derivatization to its alditol acetate, and quantified by hexose and hexosamine assays [9]. Conidial size was measured by FACS analysis (BD LSR Fortessa) of conidia of each strain fixed with 4% paraformaldehyde. Germination kinetics assay was performed by inoculating 1x10^5^ conidia per well in a 6-well plate, and incubating at 37°C, 5% CO_2_. At every hour, the number of germinated fungi was counted. For hyphal growth measurements, 1x10^6^ conidia of indicated strains were inoculated on agar plates and incubated at 37°C. Thallus diameter was measured daily. Biofilm adherence was assessed on 24h grown hyphae of indicated strains by rigorously washing and staining with 0.1% crystal violet for visualization, as previously described [10]. For scanning electron microscopy (SEM), fluorescein labeled soybean agglutinin (FITC-SBA) lectin binding, and Fc-dectin-1 binding experiments, fungi were grown for the indicated times in phenol red-free RPMI at 37°C, 5% CO_2_ incubation, fixed with either 2.5% gluteraldehyde or 4% PFA, and further processed [10]. Briefly, for SEM, samples were sequentially dehydrated in ethanol, critical point dried, coated in Au-Pd, and imaged at 20,000X magnification (Hitachi, Inc, or FEI Company), as previously described [10]. For FITC-SBA lectin or Fc-dectin-1 binding, fungi were grown in 96-well opaque plates (Thermo Fisher Scientific, Inc.) or clear-bottom plates (Corning, Inc.) for 9–12 h, and immunostained with FITC-SBA (Vector Labs, Inc.), or Fc-dectin-1 (a generous gift from Dr. G.D. Brown, Aberdeen, UK) followed by FITC labeled anti-human IgG, FCγ fragment specific (Jackson ImmunoResearch Laboratories, Inc.). Fluorescence of labeled samples was imaged by confocal microscopy (Olympus, Inc.) or relative fluorescence measured at 495 nm excitation and 515 nm emission using Spectramax fluorimeter (Molecular Devices, LLC) or Infinite M1000 (Tecan, Inc.).

### Virulence studies

For virulence studies in non-neutropenic mice, BALB/c or C57BL/6 mice, 6–8 weeks old, were immunosuppressed with 5 doses of 10 mg of cortisone acetate per mouse (Sigma-Aldrich), administered subcutaneously every other day starting on day -4 relative to infection [46]. For studies in neutropenic mice, 6–8 weeks old C57BL/6 mice were treated on days -2 and +3 with 250 mg/kg of cortisone acetate (Sigma-Aldrich) intraperitoneally, and 250 mg/kg on day -2 and 200 mg/kg on day +3 of cyclophosphamide (Western Medical Supply). To prevent bacterial infection, enrofloxacin was added to the drinking water. For studies in an NADPH oxidase-deficient host, 8–12 week old male and female *gp91* ^*phox*^ deficient mice lacking functional NADPH oxidase were used. All mice were infected intranasally with 1x10^6^ conidia from *A*. *nidulans*, An-Uge3, *A*. *fumigatus* or An-UgeB strains or treated with PBS alone as uninfected controls. Mice were monitored for a period of 2 weeks for signs of illness, and moribund animals were euthanized or were sacrificed 4 days after infection for determination of fungal burden and inflammation markers. At the time of sacrifice, bronchoalveolar lavage (BAL) fluid was collected and then lungs were harvested and homogenized [10]. To determine pulmonary leukocyte recruitment, Balb/c mice infected with the indicated fungal strains were sacrificed 4 days after infection and lungs were removed for collagenase (Sigma-Aldrich) digestion. Excised lungs were washed in PBS, minced, and digested for 1 hour at 37°C in RPMI (Wisent) and 150 U/mL collagenase (Sigma-Aldrich) supplemented with 5% fetal bovine serum (Wisent). After digestion, cells were washed and stained as mentioned, and post-fixed in 2% PFA. Cells from BAL fluid or digested lungs were labeled with CD45, CD11b, CD11c, and Ly6G (BD Biosciences), stained with Cell Viability Stain (eBiosciences), and counted using LSR Fortessa cell analyzer (BD Biosciences) and analyzed using FlowJo VX software (Tree Star). Total leukocytes were gated as CD45^+^ cells, while neutrophils were gated as CD11b^high^, CD11c^low^, and Ly6G^+^ cells. For histopathology studies, lungs from a subset of immunosuppressed BALB/c mice were fixed in formalin. Thin sections were stained with hematoxylin and eosin (H&E) or periodic acid Schiff (PAS) to visualize fungal elements. Caspase-3 immunohistopathology was performed using an anti-capsase-3 antibody (Institute for Research in Immunology and Cancer, Montreal, Canada). Immunohistochemistry staining with an anti-GAG antibody (Fontaine et al., 2011) was performed to detect GAG in pulmonary sections (Institute for Research in Immunology and Cancer, Montreal, Canada). Pulmonary fungal burden was measured by determining relative galactomannan levels by EIA (BioRad) using an internal standard [10]. Myeloperoxidase activity (Hycult Biotech), or cytokine production of TNF-α (eBiosciences, Inc.), IL-1RA (R&D Systems, Inc), or IL-1β (Sigma-Aldrich) were measured by commercial EIA following the manufacturer’s instructions. All procedures involving mice were approved by the Los Angeles Biomedical Research Institute Animal Use and Care Committee; the Montana State University Animal Care Facility; or the McGill University Animal Care Committee, and followed the National Institutes of Health guidelines for animal housing and care and the guidelines established by the Canadian Council on Animal Care.

### Morphometric analysis

PAS stained lung sections were digitally scanned at up to 400X magnification (Goodman Cancer Center, McGill University). Fungal lesions were identified by visual inspection and lesion size and distance from airways were calculated using Spectrum (Apio, Inc) software. To ensure unbiased data collection, readers were blinded to strain identity.

### Neutrophil damage experiments

Indicated fungal strains were grown at 37°C, 5% CO_2_ for 6–9 h in either Brian medium or Iscove’s Modified Dulbecco Medium (IMDM) adjusted to pH 6.5 (Life Technologies, Inc.) in a 24-well tissue culture treated plate (Corning, Inc.). For co-incubation experiments with neutrophils, blood samples obtained from healthy donors or from an individual with granulomatous disease were purified to obtain polymorphonuclear cells (PMN), as previously described [47]. Briefly, white blood cells were separated using Ficoll gradation, followed by dextran sedimentation, and red blood cell lysis. Neutrophils isolated from donors were added to wells with young hyphae and co-incubated for 12 h. After 12 h co-incubation, wells were washed with PBS, and remaining neutrophils were lysed with distilled, endotoxin-free water (Fisher, Inc.). To quantify hyphal injury, metabolic activity of fungi was measured by reduction of the tetrazolium reagent XTT (Bioshop, Inc.), as previously described [48]. To inhibit NADPH-oxidase activity, neutrophils were incubated with 25 μM diphenyleneiodonium (Sigma-Aldrich) for 1 h and washed twice in medium prior to co-incubation with fungi. To test the ability of DNAse to disrupt neutrophil extracellular traps, 10 Units of micrococcal nuclease (Sigma-Aldrich) were added to each well at the time of co-incubation of hyphae and neutrophils. To ascertain the effects of corticosteroid on neutrophil function in vitro, neutrophils were treated with 10μM dexamethasone (Sigma-Aldrich) for 30 minutes prior to co-incubation. Effect of dexamethasone treatment on neutrophil function was measured by fungal killing quantified by reduction of XTT and normalized to the metabolic activity of dexamethasone treated hyphae for each strain.

To assess non-oxidative hyphal killing, 6–9 h grown young hyphae of indicated strains were incubated with neutrophil lysates for 12 h, followed by XTT reduction assay, as previously described [48]. Neutrophil lysates were prepared by subjecting the cells through a freeze-thaw cycle, re-suspending in Brian medium pH 5.4 supplemented with 2% protease-free bovine serum albumin (Bioshop, Inc.). Neutrophils were incubated with 25 μM DPI (Sigma, Inc.) for 1 h for the preparation of DPI-treated lysates. After mechanical disruption lysates were separated from cell debris by centrifugation. The multiplicity of infection used for all assays, including PMN lysates, was 1:100 (conidia to neutrophil). All procedures involving human subjects were approved by the McGill University Research Ethics Board.

To test susceptibility to reactive oxygen species, hyphae of the indicated strains were incubated for 12 h with various concentrations of t-butyl peroxide (Sigma-Aldrich) ranging from 0 to 20 mM. Hyphal killing by t-butyl peroxide was measured by XTT reduction assay.

NET formation by neutrophils was detected by live imaging using confocal microscopy. Differentiated HL-60 cells were co-incubated for 10–12 h with 6–9 h young hyphae of indicated strains grown on coverslips. The coverslips were placed on slides containing 6–10 μL of phenol red-free RPMI (Wisent, Inc.) containing 5 μg/mL of propidium iodide (Invitrogen, Inc.) and imaged at an excitation wavelength of 543 nm laser using an Olympus Fluoview confocal microscope (Olympus, Corp.). To ensure consistency between replicate imaging, all image acquisitions were performed at room temperature, within 10–15 minutes.

### Statistical analysis

For all mouse lung data analysis, unless indicated on the legend, the Mantel-Cox log-rank test with pairwise comparison using Bonferroni correction was applied. For non-parametric data with multiple comparisons, the Krustal-Wallis rank analysis, was performed, followed by Dunn’s test for pairwise comparison. For all other data analysis, statistical significance was determined by applying one-way ANOVA, partitioned with pairwise comparison using Tukey’s test, and Bonferroni correction applied where applicable. All statistical analyses were performed either with Prism (GraphPad, Inc.) or SAS (SAS Institute, Inc.) with significance determined at *p* < 0.05. For all statistical analysis, n.s. denotes statistically no significant differences found.

## Supporting Information

S1 Fig Additional characterization of the *Aspergillus* species GAG production and surface morphology. (A) Total secreted GAG normalized to biomass for the indicated species grown in Brian medium. (B) Cell wall-associated GalNAc as detected by staining with FITC-conjugated soybean agglutinin (SBA) and quantified by fluorometry. (C) Formation of adherent biofilms on tissue culture-treated polystyrene plates by the indicated *A*. *fumigatus* and *A*. *nidulans* strains. After 24 hours growth, biofilms were washed and visualized by crystal violet staining.
(PDF)

S2 Fig Additional characterization of the *A*. *nidulans* overexpression mutant phenotypes. (A) Cell wall GalNAc detection by staining with FITC-conjugated soybean agglutinin (SBA). SBA binding to mature hyphal mats of the indicated species was quantified by fluorometry at 495 nm. (B) Relative expression of *uge3* in wild-type *A*. *nidulans* and *A*. *fumigatus* during growth in Brian medium as measured by real-time RT-PCR. (C) Germination of *A*. *nidulans* wild-type or the An-Uge3 strain in Brian medium over the indicated time period. (D) Conidia size of the indicated strains quantified using flow cytometry. FSC indicates forward scatter. Color scheme is as follows: yellow is wild-type *A*. *nidulans*, cyan is the An-Uge3 strain, and red is the An-UgeB strain. (E) Efficiency of primers for amplification of the indicated genes relative to the expression of reference gene *Tef1* in the respective *Aspergillus* species. (F) Detection of β-1,3-glucan exposure on the surface of hyphae by immunostaining with Fc-dectin-1 antibody by confocal microscopy. Images were acquired using a 488 nm laser and detected by confocal microscopy at 600X magnification. Scale bar represents 10 μm. (G) Hyphal growth as measured by radial growth of the indicated strains on Aspergillus minimal media plates. For all panels: Data are represented as mean +/- SEM and * indicates a significant difference between *A*. *nidulans*, and the An-Uge3 overexpression strain, p<0.05 by Kruskal-Wallis test with Dunn’s test for pairwise comparison.
(PDF)

S3 Fig Additional characterization of host response. (A) Percent leukocytes from total CD45^+^ cells in BAL fluid from Balb/c mice 4 days after infection with the indicated strains or PBS control as measured by CD45 detection using flow cytometry. (B) Percent neutrophils from total CD45^+^ cells in BAL fluid from of Balb/c mice 4 days after infection with the indicated strains or PBS control as measured by Ly6G^+^, CD11b^high^, and CD11c^low^ detection using flow cytometry. (C) Myeloperoxidase activity in lungs of Balb/c mice infected with the indicated strains or PBS control. (D-G) Total lung cytokine concentrations in Balb/c mice after 4 days of infection with the indicated strains or PBS control. Cytokines were measured by commercial EIA for (D) TNF-α, (E) IL-1RA, (F) IL-17, or (G) IL-1β. (H) Percent viable total leukocytes (identified as in A) as measured by viability staining using flow cytometry. (I) Percent viable neutrophils (identified as in B) as measured by viability staining using flow cytometry. (J) H&E stained lung sections from Balb/c mice infected with the indicated strains or PBS control. No significant differences in nuclear fragmentation of leukocytes were observed. For panels A-G: Data are represented as median with interquartile ranges and * indicates a significant difference between uninfected mice and mice infected with *A*. *nidulans* and the An-Uge3 overexpression strain, p<0.05 by Krustal-Wallis test with Dunn’s test for pairwise comparison.
(PDF)

S1 Table List of primers. (PDF)
