# Supplementary figures and images for: The Fungal Exopolysaccharide Galactosaminogalactan Mediates Virulence by Enhancing Resistance to Neutrophil Extracellular Traps
Source: PLoS Pathog. 2015 Oct 22;11(10):e1005187. doi: 10.1371/journal.ppat.1005187 (PMC4619649; doi:10.1371/journal.ppat.1005187)

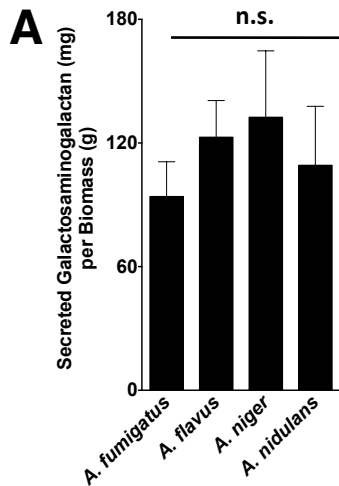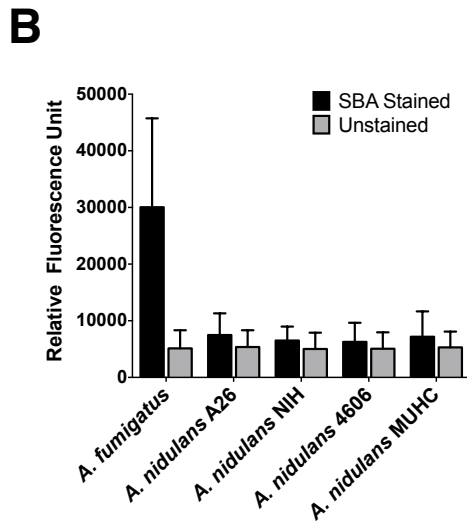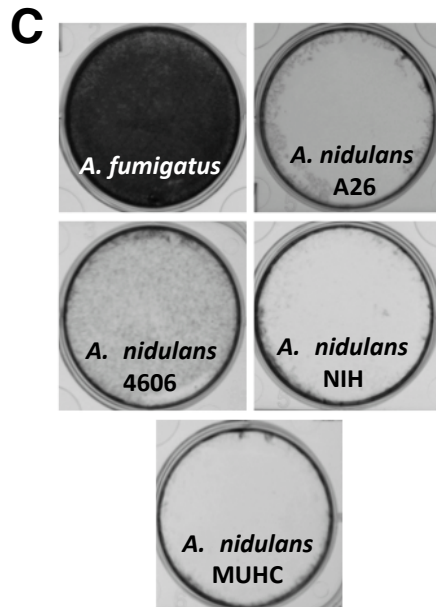

Supplement: S1 Fig — (A) Total secreted GAG normalized to biomass for the indicated species grown in Brian medium. (B) Cell wall-associated GalNAc as detected by staining with FITC-conjugated soybean agglutinin (SBA) and quantified by fluorometry. (C) Formation of adherent biofilms on tissue culture-treated polystyrene plates by the indicated A. fumigatus and A. nidulans strains. After 24 hours growth, biofilms were washed and visualized by crystal violet staining. (PDF) [file ppat.1005187.s001.pdf]

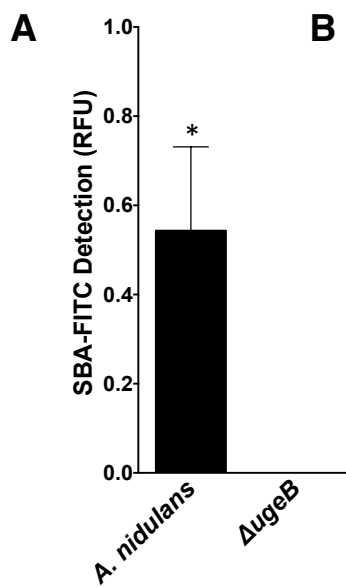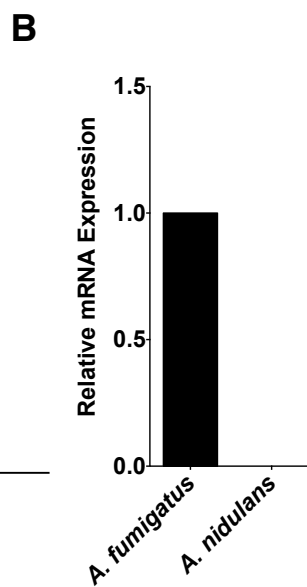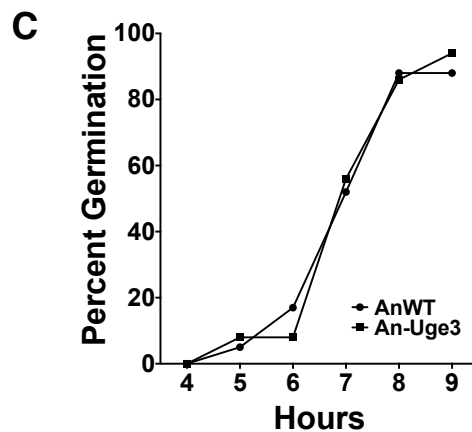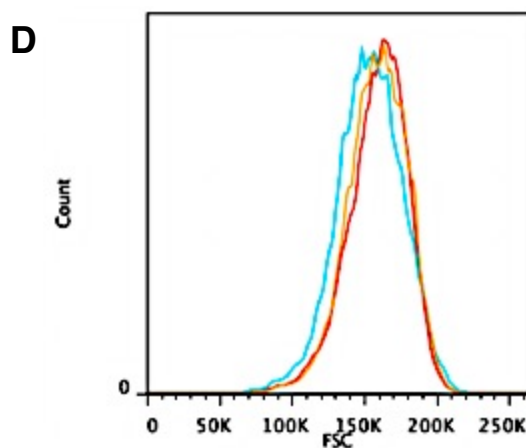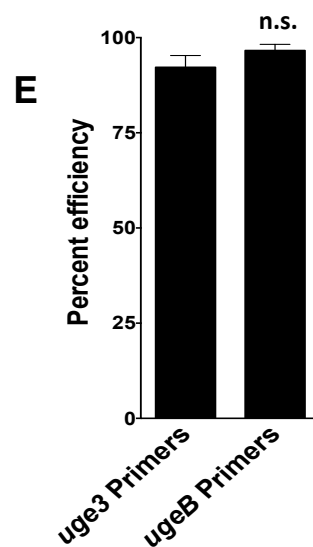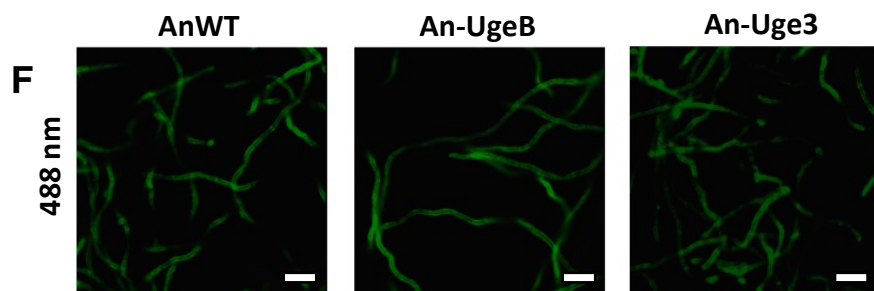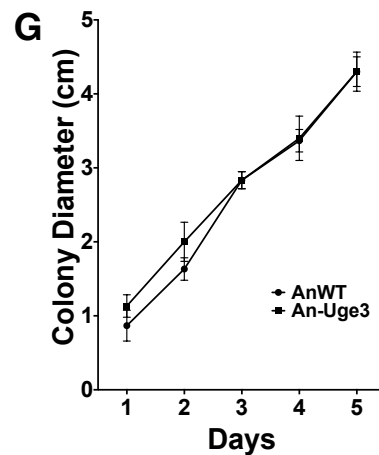

Supplement: S2 Fig — (A) Cell wall GalNAc detection by staining with FITC-conjugated soybean agglutinin (SBA). SBA binding to mature hyphal mats of the indicated species was quantified by fluorometry at 495 nm. (B) Relative expression of uge3 in wild-type A. nidulans and A. fumigatus during growth in Brian medium as measured by real-time RT-PCR. (C) Germination of A. nidulans wild-type or the An-Uge3 strain in Brian medium over the indicated time period. (D) Conidia size of the indicated strains quantified using flow cytometry. FSC indicates forward scatter. Color scheme is as follows: yellow is wild-type A. nidulans, cyan is the An-Uge3 strain, and red is the An-UgeB strain. (E) Efficiency of primers for amplification of the indicated genes relative to the expression of reference gene Tef1 in the respective Aspergillus species. (F) Detection of β-1,3-glucan exposure on the surface of hyphae by immunostaining with Fc-dectin-1 antibody by confocal microscopy. Images were acquired using a 488 nm laser and detected by confocal microscopy at 600X magnification. Scale bar represents 10 μm. (G) Hyphal growth as measured by radial growth of the indicated strains on Aspergillus minimal media plates. For all panels: Data are represented as mean +/- SEM and * indicates a significant difference between A. nidulans, and the An-Uge3 overexpression strain, p<0.05 by Kruskal-Wallis test with Dunn’s test for pairwise comparison. (PDF) [file ppat.1005187.s002.pdf]

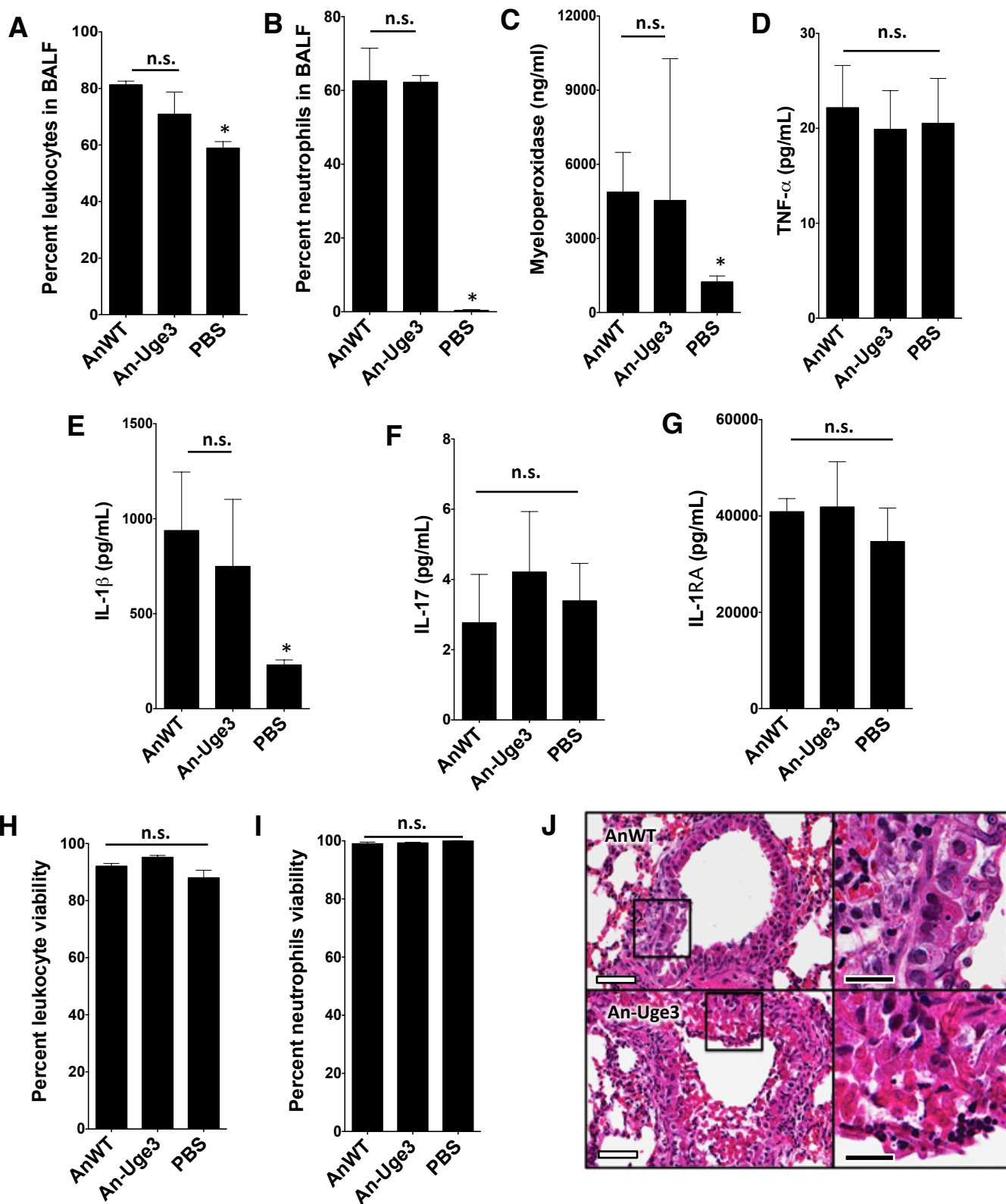

Supplement: S3 Fig — (A) Percent leukocytes from total CD45+ cells in BAL fluid from Balb/c mice 4 days after infection with the indicated strains or PBS control as measured by CD45 detection using flow cytometry. (B) Percent neutrophils from total CD45+ cells in BAL fluid from of Balb/c mice 4 days after infection with the indicated strains or PBS control as measured by Ly6G+, CD11bhigh, and CD11clow detection using flow cytometry. (C) Myeloperoxidase activity in lungs of Balb/c mice infected with the indicated strains or PBS control. (D-G) Total lung cytokine concentrations in Balb/c mice after 4 days of infection with the indicated strains or PBS control. Cytokines were measured by commercial EIA for (D) TNF-α, (E) IL-1RA, (F) IL-17, or (G) IL-1β. (H) Percent viable total leukocytes (identified as in A) as measured by viability staining using flow cytometry. (I) Percent viable neutrophils (identified as in B) as measured by viability staining using flow cytometry. (J) H&E stained lung sections from Balb/c mice infected with the indicated strains or PBS control. No significant differences in nuclear fragmentation of leukocytes were observed. For panels A-G: Data are represented as median with interquartile ranges and * indicates a significant difference between uninfected mice and mice infected with A. nidulans and the An-Uge3 overexpression strain, p<0.05 by Krustal-Wallis test with Dunn’s test for pairwise comparison. (PDF) [file ppat.1005187.s003.pdf]
