# Supplementary material for: The Fungal Exopolysaccharide Galactosaminogalactan Mediates Virulence by Enhancing Resistance to Neutrophil Extracellular Traps
Source: PLoS Pathog. 2015 Oct 22;11(10):e1005187. doi: 10.1371/journal.ppat.1005187 (PMC4619649; doi:10.1371/journal.ppat.1005187)
Supplement: S1 Table — (PDF) [file ppat.1005187.s004.pdf]

**Primer name**	**Target**	**Sequence**
Uge3-gfp-F	pUge3-OX plasmid	CATCACCCCATGGATATCATGGACAGCACCAG
Uge3-gfp-R	pUge3-OX plasmid	CATCGCGGCCGCGATATCAGTAGATAACCCACT
UgeB-gfp-F	ugeB	CATCACCCCATGGATATCATGGATGGATAGTCCTCGAC
UgeB-gfp-R	ugeB	AAGTGGATCCACTAGTTCATAAAGTAACACCGCTAA
uge3-RT-FWD.F2	uge3	GCTGTTAGCCTCCCAGTACC
uge3-RT-REV.F2	uge3	GGACTTGGTCGTACCCCAT
AfTef1-RT-FWD	*A. fumigatus* tef1	CCATGTGTGTCGAGTCCTTC
AfTef1-RT-REV	*A. fumigatus* tef1	GAACGTACAGCAACAGTCTGG
AfgpdA-RT-FWD	*A. fumigatus* gpdA	GGCATTGTTGAGGGTCTCAT
AfgpdA-RT-REV	*A. fumigatus* gpdA	ACGTTGGAGGTAGGAACACG
ugeB-RT-FWD.Q2	ugeB	TTCAACTTGGGAACTGGGCG
ugeB-RT-REV.Q2	ugeB	CGATCTGGTAGCAACGGCAA
AnidTef1-RT-FWD	*A. nidulans* tef1	TCCAGACCCCCAAGTATGAG
AnidTef1-RT-REV	*A. nidulans* tef1	ACCGGAAGCGATGATAAGG
AngpdA-RT-FWD	*A. nidulans* gpdA	GGCATTGTTGAGGGTCTCAT
AngdpA-RT-REV	*A. nidulans* gpdA	ACGTTGGAGGTAGGAACACG
